# Antioxidants, Hormetic Nutrition, and Autism

**DOI:** 10.2174/1570159X21666230817085811

**Published:** 2024-09-01

**Authors:** Sergio Modafferi, Gabriella Lupo, Mario Tomasello, Francesco Rampulla, Marialaura Ontario, Maria Scuto, Angela Trovato Salinaro, Antonio Arcidiacono, Carmelina Daniela Anfuso, Maria Legmouz, Fatima-Zahra Azzaoui, Agostino Palmeri, Sestina Spano', Francesca Biamonte, Gaetano Cammilleri, Tilman Fritsch, Alena Sidenkova, Edward Calabrese, Uwe Wenzel, Vittorio Calabrese

**Affiliations:** 1 Department of Biomedical and Biotechnological Sciences, University of Catania, Catania, 950125, Italy;; 2 Department of Biologie, Laboratory of Biologie and Health, Faculty of Science, Ibn Tofail University, Kenitra, Morocco;; 3 Food Department, Istituto Zooprofilattico Sperimentale della Sicilia, via Gino Marinuzzi, 3 90129, Palermo, Italy;; 4 NAM-Institute, Salzburg, Salzburg, A-5020, Austria;; 5 Department of Psychiatry, Ural State Medical University, Ekaterinburg, Russia;; 6 Department of Environmental Health Sciences; Morrill I, N344, University of Massachusetts, Amherst, MA, 01003, USA;; 7 Institut für Ernährungswissenschaft, Justus Liebig Universitat Giessen, Germany

**Keywords:** Autism spectrum disorders, vitagenes, antioxidants, hormesis, *C. elegans*, mushrooms

## Abstract

Autism spectrum disorder (ASD) includes a heterogeneous group of complex neurodevelopmental disorders characterized by atypical behaviors with two core pathological manifestations: deficits in social interaction/communication and repetitive behaviors, which are associated with disturbed redox homeostasis. Modulation of cellular resilience mechanisms induced by low levels of stressors represents a novel approach for the development of therapeutic strategies, and in this context, neuroprotective effects of a wide range of polyphenol compounds have been demonstrated in several *in vitro* and *in vivo* studies and thoroughly reviewed. Mushrooms have been used in traditional medicine for many years and have been associated with a long list of therapeutic properties, including antitumor, immunomodulatory, antioxidant, antiviral, antibacterial, and hepatoprotective effects. Our recent studies have strikingly indicated the presence of polyphenols in nutritional mushrooms and demonstrated their protective effects in different models of neurodegenerative disorders in humans and rats. Although their therapeutic effects are exerted through multiple mechanisms, increasing attention is focusing on their capacity to induce endogenous defense systems by modulating cellular signaling processes such as nuclear factor erythroid 2 related factor 2 (Nrf2) and nuclear factor-kappa B (NF-κB) pathways. Here we discuss the protective role of hormesis and its modulation by hormetic nutrients in ASD.

## 1 INTRODUCTION

Autism Spectrum Disorder (ASD), as an heterogeneous group of neurodevelopmental disorders, is characterized by persistent impairments in social communication and limited repetitive behavior in children [1-6]. ASD results from an interplay of factors affecting certain neural circuits, oxidative stress, neuroinflammation, and mitochondrial dysfunction [7]. Epidemiological studies show high comorbidity between ASD and mitochondrial disorders (MD) [8]. Almost 80% of patients with ASD suffer from mitochondrial dysfunction and deficiencies in adenosine triphosphate (ATP) and N-acetylaspartate (NAA), together with abnormal levels of reactive oxygen species (ROS) have been found associated with mitochondrial dysfunction in ASD [9]. Disruption in mitochondrial bioenergetics correlates with low energy metabolism in the brain, impaired synaptic plasticity, and immune response. Mitochondria of children with ASD symptoms have electron transport chain activity significantly increased compared to the mitochondria of typically developing children, predisposing them to higher levels of oxidative stress. Impaired expression of proteins and activity of mitochondrial respiratory complexes have been found associated with decreased activity of pyruvate dehydrogenase, representing strong evidence of mitochondrial dysfunction occurring in the ASD brain. A decrease in pyruvate dehydrogenase activity causes insufficient pyruvate and lactate utilization by the TCA cycle, which causes insufficient ATP production in the brain, which generally, in a vicious circle, leads to severe metabolic consequences, such as oxidative stress [10]. Consistently, high levels of ROS and low concentrations of mitochondrial glutathione are found in the brains mitochondria of children with ASD [11]. In addition, mutations in the WDFY3, Ambra1, and PARK2 genes are associated with autism symptoms, mitophagy dysfunction, and reduced synaptic brain plasticity [12]. Ambra1 (rs3802890-AA/GG) and PARK2 genes are mitophagy receptors, thus, deficiency of Ambra1 leads to severe dysfunction of neuronal mitophagy [13]. Furthermore, an increase in mtDNA deletions, point mutations, or copy numbers has been detected in patients with autism [14]. All these markers of mitochondrial dysfunction correlate with unfavorable dynamics of speech functions, behavioral regression, and the severity of autism [15]. Notably, epidemiological studies indicated that perinatal exposure to endocrine disruptors might cause neurodevelopmental disorders in children. Environmental toxicants including phthalates, polychlorinated biphenyls, polyaromatic hydrocarbons, dioxins, heavy metals, and other major groups distributed in different pesticides, are potent inducers of oxidative damage to protein, lipids, and DNA, and are generally associated with neuroinflammation, which may contribute to secondary mitochondrial dysfunction occurring in children with ASD [16]. In this context, nutritional antioxidants targeting neuroinflammation may be beneficial in children with ASD [17, 18]. The link between nutrition and autism spectrum disorder (ASD) has provided a fresh point of view and signals that nutrition may play a role in the etiology of ASD, as well as playing an effective role in treatment by improving symptoms. Even though the therapeutic evidence of dietary interventions and their mechanisms of action are very new, they provide a promising platform for designing future treatments for alleviating ASD symptoms. Studies show a positive effect on the behavior of children with ASD with brain dysfunction and biomarkers of mitochondrial dysfunction when administered with antioxidants, carnitine, coenzyme Q10, and high doses of B vitamins (thiamine or riboflavin). The intake of antioxidants, methylcobalamin, and folic acid resulted in increased GSH levels and potentially improved mitochondrial function [18]. Sulforaphane (SFN) is a dietary phytochemical extracted from cruciferous vegetables. It is an effective activator of the transcription factor Nrf2 (nuclear erythroid-2 like factor-2), which can upregulate multiple antioxidants and protect neurons against various oxidative damages. It can also significantly reduce the inflammatory response and decrease the damage caused by the immune response *via* the nuclear factor-κB pathway and other pathways [19]. In addition, a positive effect of hormesis on mitochondrial dysfunction in ASD has been suggested. Empirical experience shows improvement in febrile ASD patients, leading to the upregulation of heat shock proteins (Hsps), which mediate positive changes in synaptic function. Mitjans *et al*. [20] have indicated the hormetic dose-response concept and putative mechanisms by which hormetic dose responses may be applicable to the induction of endogenous neuroprotective processes that may be of value in the treatment of ASD. The use of small redox-active molecules (sulforaphane, resveratrol, hydroxytyrosol) regulates the cellular stress response, modulates the vitagene network, helps restore redox imbalance, improves mitochondrial function, modulates the immune response, and thus prevents neuroinflammation [20]. Hormetic adaptations have been widely reported in *C. elegans* with external stressors, such as heat, hyperbaric oxygen, or chemicals that generate reactive oxygen species. Such treatments in preconditioning protocols have significantly increased resistance not only to the same challenge but also to other stressors, displaying a form of cross-tolerance [21]. Since these adaptations to stressors depend on increased expression of stress response genes, such as FOXO target genes, including genes encoding antioxidant proteins [22], these mechanistic-based adaptations are saturable, inevitably leading to premature death once the dose of the stressor exceeds the protective adaptive capacities. It is assumed, moreover, that the generation of reactive oxygen species inside mitochondria, as derived from increased mitochondrial oxygen consumption, is fundamental to the activation of the hormetic response. This response at the level of mitochondria has been widely referred as mitochondrial hormesis, or mitohormesis. Mitohormesis appears to be triggered especially by the β-oxidation of fatty acids [23], a metabolic condition induced by caloric restriction. However, although many hormetic adaptations observed as a consequence of caloric restriction are dependent on sirtuins, in *C. elegans* sir-2.1 [24], hormesis as a consequence of glucose restriction and increased β-oxidation was shown to be independent of sir2.1 but dependent on AMP-kinase [23]. Overall, genetic analyses have revealed that sir-2.1 and the FOXO-orthologue DAF-16 possess overlapping but also distinct functions regarding the regulation of lifespan in response to caloric restriction [25]. Finally, similar to the stimulation of stress response pathways by metabolic interventions, phytochemicals also activate hormetic mechanisms as evidenced by increased protection *versus* oxidative and thermal stress and extension of the lifespan of wildtype nematodes in association with an HSF-1 and SKN-1/ Nrf2 dependent increased expression of stress-protective genes such as Hsp-16.2, Hsp-6, Hsp-60 and Gst-4 [26].

## 2 *CAENORHABDITIS ELEGANS* AS A TRACTABLE MODEL TO STUDY MOLECULAR MECHANISMS UNDERLYING ASD PATHOPHYSIOLOGY

The nematode *Caenorhabditis elegans* serves as a reliable neurobiological model for the investigation of molecular mechanisms underlying neurodegenerative diseases due to the well-understood development and lineage of the nervous system [27]. The neurodevelopmental disorder autism is characterized by abnormalities in communication, social relationships, and patterns of behaviour [28]. It is well established that besides environmental factors there is also a strong genetic contribution to autism [29].

**Fig. (1):**
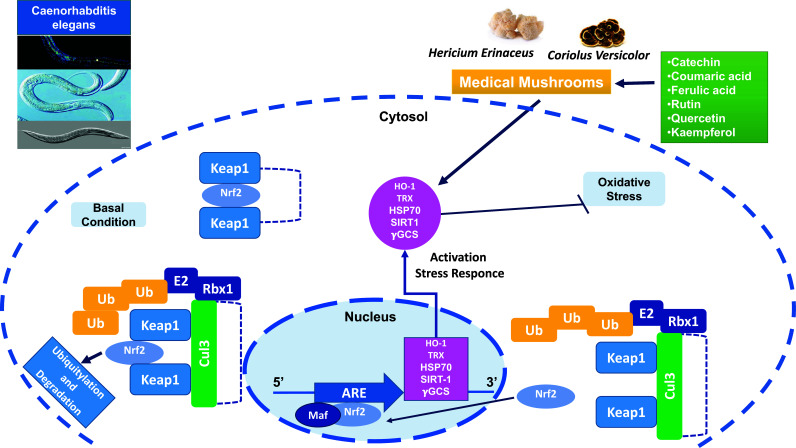
Nrf2 regulation through KEAP1 under basal and stress conditions. Under basal conditions, NRF2 localizes in the cytosol and interacts with the Cul3-Rbx1 E3 ubiquitin-ligase substrate KEAP1 that constantly primes NRF2 for ubiquitination and proteasomal degradation. Oxidative/electrophilic stress causes conformational changes of KEAP1 through the modification of cysteine residues in IVR and BTB domains leading to NRF2 dissociation. Free NRF2 enters into the nucleus where it forms dimers with small MAF proteins or other interactors and binds to the antioxidant-responsive elements (AREs) regulatory sequences of target genes, inducing their expression.

Since *C. elegans* display about 80% homology to human gene sequences and more than 42% to human disease-related genes [30], it could also provide a valuable model for the investigation of autism. This is exemplified by the investigation of *C. elegans* mutant strains for orthologues of selected human genes with relevance for autism. A food-leaving assay in these mutant strains served the evaluation of the mutant gene on the autism phenotype, based on the findings that progeny-derived social cues mediate a progeny-dependent increase in adult food-leaving behaviour (Fig. **1**) [31].

Since *C. elegans* is amenable to high-throughput technologies, this allows to describe hundreds of autism-relevant genotype-phenotype relationships, such as severe developmental delays and uncoordinated movement or subtle deficits in sensory and learning behaviours [32]. RNA-interference (RNAi) is another high-throughput technology to be exploited in *C. elegans*. Knocking down orthologues for candidate neuropsychiatric risk genes, whose variants are predicted to play a role in autism spectrum disorders, regulation of neuronal morphology, with an individual impact of single genes on dendritic branching was observed [33]. RNAi *versus* orthologous genes of the human androgen receptor gene, diminished the impairment of the gentle touch response and pharyngeal pumping, as parameters of the behavioural pattern [34]. Fetal testosterone is suggested to play a negative role in cognitive and psychological brain development in autism spectrum disorders [35]. The effects of testosterone in *C. elegans* were stable over four generations also in the absence of testosterone, demonstrating that the nematode, especially according to its very short life cycle of only a few days, provides also a valuable tool for the investigation of epigenetic effects on autism-related processes.

Numerous studies have associated autism with mutations in several genes involved in excitatory and inhibitory synapses in the mammalian brain. Consistent with this notion, several molecular pathways and behavioural phenotypes in *C. elegans* have been related to autism, owing to a series of advantages which, in combination with knowledge derived from other animal models and human researches, provide a powerful approach for understanding the molecular mechanisms and underlying aetiology of not only ASD but also other complex neurological diseases.

## 3 GUT-BRAIN AXIS AND ASD

In recent years, numerous studies have highlighted the existence of a correlation between the intestine and the brain, demonstrating that gut microbiota (GM) can influence brain functions [36]. The discovery of the gut-brain axis has laid the foundations for understanding dysfunctional neurological processes for which GM intervention would seem to play a key role. Indeed, GM could influence the nervous system by altering the immune system, modifying the metabolism, and interfering with the release of neurotransmitters [37].

It has been demonstrated that changes in the composition of GM can damage the intestinal epithelial barrier, allowing the translocation of enteric bacteria and activating immune and inflammatory reactions [38].

It is known that the inflammatory process can damage the blood-brain barrier (BBB), altering the tight junctions sealing the endothelial cells and inducing the detachment of pericytes, physiologically associated with the endothelium in an intact BBB [39].

The microbial gut-brain axis allows bidirectional communication between the GM and the brain, and its dysfunction is related to neurodegenerative and neuropsychiatric disorders such as Alzheimer's disease, bipolar disorder, depression, Huntington's disease, Parkinson's disease, and schizophrenia [40].

Numerous studies, both in animal models and in clinical studies, have demonstrated that changes in GM composition can aggravate behavioral disturbances in children with autism (ASD) [41].

The increase in the permeability of the intestinal barrier, following dysbiosis, causes the passage of intestinal bacteria and circulating lipopolysaccharides which trigger an immune response with consequent brain damage. Furthermore, even short-chain fatty acids (SCFA), metabolised by microbiota, cross the BBB and penetrate the brain where they play a dual role, regulating the synthesis of neurotransmitters, such as serotonin and dopamine [42]. Cytokines produced in the intestine can pass to the brain where the BBB is deficient and affect brain areas such as the hypothalamus. The cytokines interleukin (IL)-1 and IL-6 cause cortisol release upon stimulation of the hypothalamic-pituitary-adrenal axis, thereby regulating neuro-immune signaling responses [43]. Moreover, it has been shown that an impaired immune system could activate the inflammasome, which could lead to ASD.

SCFAs, produced by bacterial fermentation of carbohydrates, are represented by butyrate (BT), acetic acid, valeric acid, and propionic acid. In addition to having immunomodulatory properties, they contribute to the pathogenesis of ASD both by modifying mitochondrial metabolic functions, especially the Krebs cycle and carnitine metabolism, and by inducing epigenetic changes in the genes that cause ASD [44]. Among these, BT performs numerous protective functions, as it strengthens the intestinal tight junctions allowing the maintenance of an intact barrier, and also has a beneficial effect on the liver, by modulating the inflammatory response [45].

A study conducted on Chinese children with ASD demonstrated that BT-producing intestinal bacteria were present in fewer numbers while *Fusobacterium*, *Barnesiella*, *Coprobacter*, and *Actinomycetaceae* were present more in numbers and that the resulting dysbiosis induced an increase in intestinal permeability [46]. It has also been shown that BT can induce an increase in the synthesis of enzymes of the melatonergic pathway and an increase in N-acetylserotonin and melatonin [47].

The GM can also influence the immune system by modifying the contents of the exosomes released. In particular, miRNAs, encased in exosomes, can vary allowing the modulation of gene transcription in cells distant from cells that have released exosomes, including immune cells [48].

The presence of pathogenic bacteria in GM could play a key role in the onset of ASD. In particular, it has been shown that Candida albicans is twice as prevalent in the gastrointestinal tract of children with ASD than in healthy children and this could trigger autistic behavior following the production, by these bacteria, of ammonia and other toxins [49]. Despite this, the correlation between the marked presence of gut bacterial populations and ASD is controversial. While studies conducted on children with ASD have shown that the Clostridium family was 46 times greater than in the control group, correlating this presence to the onset of ASD on the other hand a statistically significant association was not found between the presence of *Clostridium* strains and severity index in autistic cases, demonstrating that gastrointestinal dysfunction should be considered as common comorbidity in ASD [50].

Numerous studies have investigated the impact of gut microbiota on immune functions in ASD. In patients with ASD, elevated amounts of inflammatory molecules such as interleukin-1 (IL-1), tumor necrosis factor (TNF), chemokine ligand 8 (CXCL8) [51], and increased gene expression of IL-18R, which is a receptor for both IL-18 and IL-37 have been found. Moreover, it has been demonstrated that gut immune cells of ASD patients secrete higher amounts of IgA than that of healthy children, suggesting gut immune abnormalities in ASD patients [52].

Fecal Microbiota Transplantation is an experimental method for treating ASD symptoms, which is used to transfer healthy bacteria from healthy participants to patients with intestinal dysbiosis, in order to rebalance the physiological GM. and alleviate gastrointestinal and neurological/behavioral symptoms in children with ASD [53].

It is difficult to get children with ASD to follow a diet as they have impaired eating behaviour, and, consequently, introduce few macro and micro nutrients, in particular less iron, cooper, docosahexaenoic and docosapentaenoic acids [54]. However, the type of food introduced into the diet is very important as it influences the GM composition in children with ASD. It has been demonstrated that children with ASD subjected to a balanced diet, particularly enriched with micronutrients, showed an improvement in autistic symptoms and behaviour [55]. For example, it has been demonstrated that a diet based on food gluten and casein-free, enriched with vitamins B6 and B12, glutathione, magnesium, carnitine, and fats improved learning, memory, and hyperactive behaviour of ASD patients [56].

## 4 HORMETIC NUTRITION

The cellular stress response is the ability of a cell to counteract stressful conditions. This phenomenon, which includes heat shock response (HSR), represents an ancient and highly conserved cytoprotective mechanism [57]. Cellular stress response requires the activation of pro-survival pathways, which produce molecules endowed with anti-oxidant and anti-apoptotic activities. Among the cellular pathways conferring protection against oxidative stress, a key role is played by the products of vitagenes. These include members of the heat shock protein (Hsp) family, such as heme oxygenase-1 and Hsp72, sirtuins, and the thioredoxin/thioredoxin reductase system 48 [58]. The cellular stress response is regulated at the transcriptional, translational, and post-translational levels by a family of heat shock transcription factors (HSFs) that are expressed and maintained in an inactive state under non-stress conditions. HSFs, essential for all organisms to survive acute or chronic stress, are also important for normal development and lifespan-enhancing pathways, and the repertoire of HSF targets has thus expanded well beyond the heat shock genes. Post-translational regulation of HSFs is emerging to integrate the metabolic state of the cell with stress biology, whereby controlling fundamental aspects of the health of the proteome and aging [59].

The vast majority of evidence demonstrating “hormetic nutrition” is based on cell culture studies where detailed concentration responses relationships showing hormetic dose responses have been widely and reproducibly reported for a large number of agents. Many of these studies have been critically assessed in agent-specific critical reviews. These studies have also included detailed assessments of underlying molecular mechanisms and detailed cell signaling pathway involvements (*e.g*. resveratrol [58], curcumin [60], ginko biloba [61], green tea [62], ginseng [63], melatonin [64], alpha lipoic acid [65], luteolin [66], Ferulic acid [67], sulforaphane [68] and others. It should be emphasized that the maximum hormetic response is typically in the 30-60% range above the control group. It is believed that this represents the limits of biological plasticity in essentially all cell types for all endpoints demonstrating hormetic dose responses. These observations have important implications since clinical trials and other epidemiological investigations find it difficult to detect responses less than about 2-3 fold greater than the control group. Thus, hormetic dose responses with excellent mechanistic understandings are well known and established in the experimental literature but are very challenging to study and prove within epidemiological settings. This is a critical issue that confronts the biomedical communities and results in many very promising drugs and other interventions being rejected, most likely as false negative findings. This situation has significant implications for translational medicine, including the possible treatment of autism that is based on the outcomes of clinical trial studies [69].

Nutritional interventions are the linkage between hormesis and ASD as noted above as “hormetic nutrition”. Despite the above challenging perspective, emerging preclinical and clinical evidence suggests that moderate dosage and/or concentration of antioxidant supplements in the diet have the potential to improve clinical symptoms of ASD by the induction of antioxidant signaling pathways (Nrf2 and related genes such as Hsp70, GSH, HO-1) in the brain of both children with ASD [70] and in a rodent model of ASD-like disorders [71]. Of relevance, a recent clinical trial demonstrated that sulforaphane supplement induces a change in urinary metabolites in particular N-methylglutamate, glutamine, hypoxanthine, serotonin, and homovanillate (HMV) and above all sphingolipid/sphingomyelin group and amino acid metabolism/gut microbiome metabolites highly associated with clinical improvements in children and young adults with ASD through an increase in antioxidant capacity including Nrf2-mediated induction of phase 2 detoxification enzymes [72].

Several phytochemicals act through the activation of transcription factor Nrf2, which after binding to the ARE (antioxidant responsive element) in the HO-1 gene, up-regulates both HO-1 and TrxR, thus counteracting pro-oxidant conditions (Fig. **1**). Nrf2 activity is regulated by the binding action of its agonist and inhibitor, Keap1. Under basal conditions, transcription factor Nrf2 is bound to a cytoplasmic repressor Keap1, which targets Nrf2 for ubiquitination and proteasomal degradation *via* association with the cullin 3-based E3 ubiquitin ligase complex. Small-molecule inducers modify highly reactive (sensor) cysteine residues of Keap1, which loses its ability to target Nrf2 for degradation. This results in the stabilization of Nrf2, binding to the ARE (in heterodimeric combinations with a small Maf transcription factor), and activation of the transcription of cytoprotective vitagenes [73]. Under basal conditions, these protective systems do not operate at maximum capacity but can be induced to higher activity levels by redox-active compounds such as antioxidants thus reducing the risks of developing malignancies and multiple chronic diseases.

Another key transcription factor is NF-κB complex which mediates a crucial role in inflammation, immunity, cell proliferation, development, survival, and apoptosis. In canonical, NF- kB binds to IkB and is retained in the cytoplasm. While the stimulus triggers a cascade of events leading to IκB phosphorylation from the B kinase inhibitor complex (IKK), NF-kB is released from I kB and translocated to the nucleus a regulate gene expression. The activity of the IKK complex and NF-kB is subject to be regulated through redox modification on cysteine. Consequently, NF-kB dysregulation has been implicated in diverse human pathologies ranging from autoimmune diseases, cancer, and neurodegenerative diseases. In particular, the transcription factor NFkB is activated in neurons in response to oxidative stress and plays a pivotal role in the adaptive response that protects the neurons against more severe oxidative stress. Notably, multiple numbers of evidence demonstrated a functional cross-talk between Nrf2 and NF-κB pathways. The first evidence of the interplay between Nrf2 and NF-κB pathways came from Nrf2 knockout (KO) mice, which exhibited a neurodegenerative phenotype The lack in Nrf2 leads to increased NF-κB activity and consequential overproduction of cytokines. The increased cytokine production induced astrogliosis, neuronal death, and demyelination of neuronal axons. On another hand, Nrf2 transcription and activity can be regulated by NF-κB, with both positive and negative effects on the target gene expression. Therefore, since both Nrf2 and NF-κB are sensitive to changes in redox homeostasis, the lack of Nrf2 led to increased oxidative and nitrosative stress, which activated NF-κB leading ultimately to amplification of cytokine production [74].

Besides this, several studies suggested that the brain's energy metabolism, mitochondrial functions, and redox balance are altered to varying degrees in brain disorders including ASD [20, 75, 76].

## 5 MEDICAL MUSHROOMS AS NRF-2 ACTIVATORS WITH POTENTIAL THERAPEUTIC POTENTIAL IN AUTISM

Mushrooms are also emerging as strong nutritional supplements for human health. Since ancient times have been a food constituent in different diet cultures and have been used as traditional medicine for thousands of years [77]. Mushrooms contain a wide range of bioactive substances such as carbohydrates (chitosans, β-glucans/lentinan, trehalose) proteins (ribosome-inactivating proteins, antifungal proteins, ubiquitin-like proteins, protease inhibitors lectins), fatty acids (linoleic acid, oleic acid and palmitic) vitamins, terpenoids (carotenoids such as β-carotene and lycopene) phenolic compounds (caffeic acid, gallic acid, cinnamic acid, melatonin, p-hydroxybenzoic acid, p-coumaric acid, and protocatechuic acid) and other molecules (such as ergothioneine and glutathione) [78]. These bioactive compounds demonstrated to exert immunomodulatory, antimicrobial, antioxidant, and anti-inflammatory actions [79]. Due to their phenolic and antioxidant contents, mushrooms have been proposed for potential treatments in a wide range of pathological conditions including neurodegenerative disorders [80, 81].

### 5.1 *Hericium erinaceus*

*Hericium erinaceus* (HE) is among the most characterized medicinal mushrooms and has been studied for its beneficial effects on human health. In particular, HE induced several antioxidant and anti-inflammatory effects in neurodegenerative disorders. Erinacines, constituents of *H. erinaceus* mycelium can pass the blood-brain barrier and exert neuroprotective functions [82]. In a mouse model of AD, HE induced a reduction of the deposition of Aβ and reduced the levels of reactive astrocytes and microglia [83]. In addition, HE can stimulate the production of nerve growth factor which modulates the activity of choline acetyltransferase and acetylcholinesterase, responsible for the dysfunction of cholinergic neurons in Alzheimer’s disease [82]. Notably, HE could exert a protective effect against oxidative stress and apoptosis induced by di(2-ethylhexyl)phthalate (DEHP), a plasticizer known to cause neurotoxicity, in pheochromocytoma 12 (PC12) cells. The protective effect was attributed to its ability to reduce intracellular levels of reactive oxygen species, preserving the activity of respiratory complexes and stabilizing mitochondrial membrane potential [84]. Furthermore, the anti-neuroinflammatory and antioxidant activity of *Hericium erinaceus* has been demonstrated in models of neuropsychiatric disorders [82, 85-88]. In a mouse model of anxiety and sleep disruption, *Hericium erinaceus* mycelium induced an antidepressant-like effect modulating BDNF/TrkB/PI3K/Akt/GSK-3β pathways and ameliorated the rodent anxiety and sleep disturbance [85]. A number of studies focused on the behavioral and physiological effects of different *H. erinaceus* extracts indepression disorder. Amycenone, a HE extract derived from the fruiting body, demonstrated antidepressant-like effects in an animal model of LPS-induced inflammation-associated depression [86]. Antidepressant and anxiolytic effects of *H. erinaceus* extracts from the fruiting bodies in adult mice were demonstrated by the reduced time spent in the peripheral region of the open field test and the reduced immobility time in both the tail suspension test and forced swim test [87]. Another study investigating the properties of an extract of HE from the mycelium, in a mouse model of depression, confirmed the capacity of HE to prevent depression-like behaviors, while an anxiolytic effect was not observed [88].

### 5.2 Coriolus Versicolor

Similarly to HE, Coriolus Versicolor (CV) is a well-known traditional food and medicinal mushroom extensively studied for its beneficial properties which include antioxidant, anti-inflammatory antimicrobial, and immunomodulatory effects [89]. Recent studies evaluated the potential role of CV in brain disorders. In a mouse model of Alzheimer’s disease CV increased the levels of two antioxidant enzymes, SOD and catalase, and reduced pro-inflammatory cytokines such as TNF-a and IL-1b; the modulation of these parameters was associated with the improvement in spatial memory [90]. Further evidence of the neuroprotective role of CV in neurodegeneration emerged in recent studies conducted in our laboratory [5, 6, 81, 90, 91]. Administration of CV biomass preparation for 30 days induced upregulation of lipoxin A4 (LXA4) predominantly in the cortex and hippocampus regions of rat brains. Induction of LXA4 led to increased levels of redox sensitive proteins involved in cellular stress response, such as Hsp72, heme oxygenase-1, and thioredoxin, suggesting a relevant impact of this nutritional intervention on the cellular stress response mechanism operating in the central nervous system [89]. LXA4 may represent a potential therapeutic target for AD-related inflammation and neurodegenerative damage, consistent with the notion that oxidative stress-driven neuroinflammation is an early pathological feature in neurodegenerative diseases. Likewise, Meniere’s disease (MD) is characterized by systemic oxidative stress. MD patients treated with CV biomass showed reduced oxidative stress markers (protein carbonyls, hydroxynonenals, F2-isoprostanes) in peripheral blood and upregulation of vitagenes (HO-1, Hsp70, Trx, sirtuin-1, and γ-GC liase) in lymphocytes, indicating a maintained response to counteract intracellular pro-oxidant status [91]. Furthermore, one of the studies evaluated the ability of CV supplementation to stimulate neurogenesis in mice hippocampus [92]. Notably, CV biomass induced a significant increase in dendritic volume, length, and branching of hippocampus immature neurons associated with higher B-catenin levels in the nucleus and cytoplasm of these cells. The increased hippocampal dendritic arborization is relevant for brain cognitive reserve and plasticity. In addition, the Wnt/β-catenin pathway may play an important role in the CV-positive effect on the differentiation of these cells [93]. The results illustrated above demonstrate HE and CV are endowed with multiple neuroprotective properties, which might counteract some molecular mechanisms underlying ASD pathophysiology. Interestingly a recent study highlighted the role of HE mycelium in promoting oligodendrocytes differentiation and production of myelin basic protein *in vitro* and *in vivo*. In particular, the study demonstrated that two bioactive components of HE, HeA, and HeS, capable to cross the brain barrier, may induce oligodendrocytes maturation and myelination, thus facilitating action potential propagation [94]. Since OL maturation, myelination, and brain connectivity were reported to be impaired in ASD [95, 96] [97], future studies should evaluate the potential of HE to regulate such pathological mechanisms in ASD. Moreover, HE mycelium enriched with Erinacine A was reported to enhance nerve growth factor (NGF) activity to promote neurite outgrowth [98]. The latter is a key process in brain development which is impaired by both environmental and genetic risk factors associated with ASD, as reported in various studies [99, 100]. Similarly, as mentioned above, CV might have an impact on the Wnt/β-Catenin pathway and dendritic arborization which are dysregulated in ASD [93, 101]. Therefore HE and CV supplementation could induce beneficial effects on brain development and neurodevelopmental disorders. Finally, as NRF2 activators, these mushrooms may promote resilience during brain development by enhancing the redox potential through neurohormetic mechanisms, such as vitagenes upregulation [102, 103]. However, there is a lack of studies that investigated HE and CV effects on ASD pathophysiology. In light of the evidence discussed above, we hypothesize that these compounds may exert potential therapeutic actions in ASD and should be tested using innovative *in vitro* platforms.

## 6 REDOXOMICS IN ASD

**Fig. (2):**
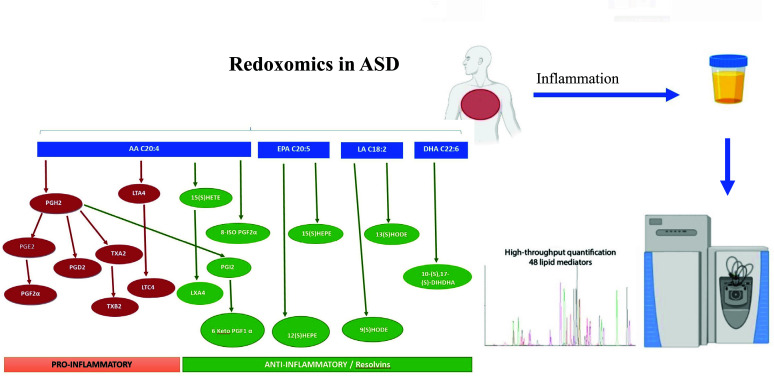
Lipid mediators, highly bioactive compounds synthesized from polyunsaturated fatty acids (PUFAs), have a fundamental role in the initiation and signaling of the inflammatory response. Oxylipin biosynthesis cascade of pro-inflammatory (red) and antinflammatory (green) stable degradation products are indicated. The development of mass spectrometry platforms enabling quantification of diverse lipid species in human urine is of crucial importance to understand metabolic redox homeostasis in normal as well as pathophysiological conditions, such as in ASD.

Quantification of diverse lipid species in human urine is of considerable importance in normal and pathophysiological conditions to study redox metabolic homeostasis. This identification is possible by mass spectrometry platforms. Oxidation of polyunsaturated fatty acids allows the synthesis of bioactive lipids: linoleic acid, dihomo-γ-linolenic acid, arachidonic acid, eicosapentaenoic acid, and docosahexaenoic acid generate bioactive lipids, endowed with powerful anti-inflammatory effects [104]. The development of mass spectrometry platforms enabling the quantification of diverse lipid species in human urine is of crucial importance to comprehend the metabolic redox homeostasis in normal as well as pathophysiological conditions, such as in ASD. In addition, in light of what has been outlined above on the therapeutic potential of mushroom phenolic compounds for this pathology, it follows that further studies should be undertaken to evaluate the potential of these compounds in regulating pathological mechanisms in ASD (Fig. **2**). In addition to this, mapping the presence of phenol compounds (or their metabolites) in human tissues with the redoxomics approach, it is crucial to understand the degree of absorption, metabolism, conjugation, and excretion of polyphenols, since it has been found to present high variability, both within and between individuals, as well as polyphenol bioactivities is are also a factor of variability, in itself [105]. For this reason, the use of innovative *in vitro* platforms is essential with a high-throughput sensitivity matrix profile. When mass spectrometry is used for the identification of the separated analytes, the choice of the analyte ionization mode turns out to be crucial to unambiguously identify molecular ions and their fragments. In particular, for the characterization of anthocyanin glycosides in the presence of the corresponding flavonol glycosides, the acquisition of MS spectra in the negative ionization mode has been shown a valuable tool for differentiating anthocyanins from non-anthocyanin polyphenols. Another important issue in the UPLC-MS analysis of polyphenols is their quantification. The content of these metabolites in a given extract can be determined by UPLC-ESI-MS/MS analysis. In this case, the triple quadrupole MS detector is operated in MRM (Multiple Reaction Monitoring) scan mode, which ensures high sensitivity and selectivity of the analysis [106]. This technique allows you to quickly distinguish the compounds that have the same parent ions (same molecular weight) but give different fragment ions. Combining a specific mass of a precursor ion with the equally specific mass of one of its product ions is generally an unambiguous and sensitive method of selectively tracking and quantifying a compound of interest. Since two stages of mass selection are utilized, MRM assays are particularly useful for the specific analysis of target compounds in complex mixtures and bio-matrices [106]. A further aspect to consider when tracing polyphenols in human tissues concerns the extraction stage from these tissues before their qualitative and/or quantitative analysis. Unlike the analysis of polyphenols in plant extracts or foods, standardized procedures for the preparation, extraction, and analysis of these compounds in human tissues are not yet available [107]. Of the methods offered in the current literature [108], each may have specific advantages toward the extraction and detection of certain polyphenolic compounds and this must be accounted for prior to analysis. To this end, iPSCs-derived 3D organoids look well to be ideal platforms on which to carry out investigations that can be profitably standardized, as well as (see below) the use of alternative experimental platforms, such as *C. elegans* models of ASD.

## CONCLUSION AND FUTURE DIRECTIONS

While computational biology continues to improve with respect to predictions and molecular modeling, the differences between *in silico* and *in vivo* analysis remain substantial. Invertebrate *in vivo* model systems represent technically advanced, experimentally mature, high-throughput, efficient, and cost-effective resources for investigating disease. Autism spectrum disorder (ASD) is a neurodevelopmental disorder characterised by a triad of behavioural impairments and includes disruption in social behaviour. Investigations are strongly sustained by experimental approaches that permit tractable investigation of the underlying genetic basis of circuits that control behaviours related to biological domains that are neuro-atypical in autism. The model organism *C. elegans* provides an experimental platform to investigate the effect of genetic mutations on behavioural outputs including those that impact social biology *C. elegans* has a high reproductive capacity and a life cycle of about three weeks under standard culture conditions; this limited adult life span makes *C. elegans* a suitable tool for high-throughput compound screening approaches. The *C. elegans* genome presents more than 42% of human disease-related genes and displays about 80% homology to human sequences [109]. Functional studies of relating or corresponding human genes can be accomplished by RNA interference (RNAi) or either with various mutants available [110]. Moreover. in the absence of endogenous homologs, *C. elegans* can be transgenically manipulated to express human disease-associated genes in specific cell types, including neurons [111, 112]. Transparency of *C. elegans* allows the *in vivo* visualization of neuronal function by expression offluorescent protein reporters in free form or attached to transgenic proteins [113]. Finally, even the co-expression of pathogenic proteins is possible for the co-expression of β-amyloid and tau, involved in the pathogenesis of Alzheimer´s disease [114], or for α-synuclein and β-amyloid in the pathogenesis of Lewy-body dementia [115].

Besides this, nowadays we have the unique opportunity of studying neural cells differentiated from human induced pluripotent stem cells (hiPSCs), testing hypotheses previously originated in small animals, and suggesting new ones specific for humans.

New technologies based on induced pluripotent stem cells (iPSC) and three-dimensional culture systems as brain organoids represent ideal platforms for modelling brain developmental disorders and offer unprecedented opportunities for drug discovery studies, as well gene-environment interactions operating in the mechanistic determinism of complex psychiatric diseases [116, 117]. The morphofunctional basis of ASD is characterized by an atypical trajectory of brain maturation, impaired neurogenesis, synaptogenesis, and an imbalance in the excitatory and inhibitory systems of the CNS [118]. These pathological changes appear at different stages of brain maturation as the result of multifactorial gene-environmental influences. Human brain organoids are self-organizing three-dimensional cell aggregates able to recapitulate brain-specific cytoarchitecture [119], summarizing multiple brain processes including neurogenesis, gliogenesis, synaptogenesis, cell migration, gyrification of the cerebral cortex and cell-cell, and cell-matrix interactions [120]

iPSC allows the generation of personalized brain models from ASD patients with specific genetic backgrounds. Alternatively, the genome of iPSC can be modified using gene engineering technology to generate isogenic iPSCs with gene correction and genetic disruption [121]. Besides minimal patterning of brain organoids composed of multiple regional identities, the addition of patterning factors, such as modifiers of Smad, Wnt, and Shh signaling, allowed to drive organoid differentiation towards specific brain regions. Cortical organoids have been suggested to be appropriate for modelling mechanisms underlying ASD including defects in cortical connectivity and neural migration to the cerebral cortex [122]. Telencephalon organoids generated from idiopathic ASD patients have shown deficits in neuronal migration, aberrant cell proliferation, abundant synaptogenesis, increased branching of neurons, high activity of ion channels, and FOXG1-dependent overproduction of GABAergic neurons. Notably, the shift towards GABAergic neurons induced by FOXG1 was positively correlated with the severity of ASD symptoms [123]. Prevailing GABAergic interneuron differentiation was also observed in organoids carrying a heterozygous mutation in CHD8, a high-risk gene for ASD. CHD8 haploinsufficiency led to a significant enlargement of cerebral organoids (*in vitro* correlate of patients' macrocephaly) and disrupted differentiation dynamic of excitatory and inhibitory neurons, with a longer phase of excitatory neuron progenitor proliferation, and accelerated production of inhibitory neurons [124]. CHD8-mutated organoids have been further employed to investigate gene-environment interactions and revealed higher susceptibility to the toxic effect of chlorpyrifos. Perturbations in metabolic and neurotransmitter systems involved in ASD and excitatory/inhibitory imbalance have been identified compared to control organoids [99]. Thus, IPSC-based models can be utilized for the identification and validation of candidate pharmacological compounds with a high impact on ASD pathogenesis.

Finally, organ-on-chip technology, consisting of engineered microchips, is a powerful innovation able to provide tissue-tissue interfaces, with the integration of circulating immune cells, connective tissue cells, and a complex microbiome [125]. We suggest that iPSCs 3D organoid and organ-on-chip systems represent the ideal platforms for studying ASD in human-relevant models and testing efficiently the potential beneficial effects of promising compounds as those discussed in this review.

## LIST OF ABBREVIATIONS

ASD: Autism Spectrum Disorder
ATP: Adenosine Triphosphate
BBB: Blood-brain Barrier
GM: Gut Microbiota
Hsp: Heat Shock Protein
HSR: Heat Shock Response
IL-1: Interleukin-1
MD: Mitochondrial Disorders
NAA: N-acetylaspartate
RNAi: RNA-interference
ROS: Reactive Oxygen Species
SFN: Sulforaphane
TNF: Tumor Necrosis Factor
